# *catena*-Poly[[trimethyl­tin(IV)]-μ-phenyl­seleninato-κ^2^ *O*:*O*′]

**DOI:** 10.1107/S1600536810054243

**Published:** 2011-01-12

**Authors:** Mengjie Guo, Jing Ru, Rufen Zhang

**Affiliations:** a College of Chemistry and Chemical Engineering, Liaocheng University, Shandong 252059, People’s Republic of China

## Abstract

In the title polymeric coordination compound, [Sn(CH_3_)_3_(C_6_H_5_O_2_Se)]_*n*_, the Sn^IV^ atom has a distorted trigonal–bipyramidal geometry, with two O atoms of two symmetry-related bridging phenyl­seleninate anions in axial positions and three methyl groups in equatorial positions. In the crystal, the complex exhibits a chain structure parallel to the *b* axis.

## Related literature

For the applications and biological activity of organotin compounds, see: Dubey & Roy (2003 ▶). For a related structure, see: Chandrasekhar *et al.* (1992 ▶).
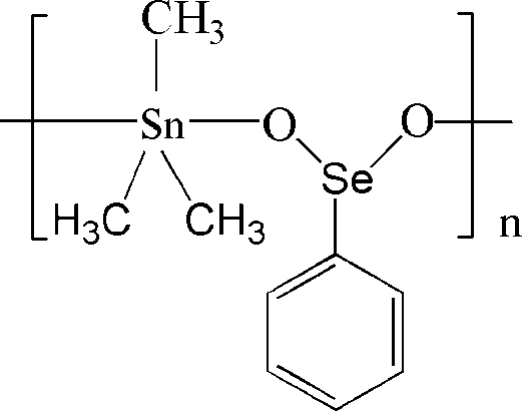


## Experimental

### 

#### Crystal data

[Sn(CH_3_)_3_(C_6_H_5_O_2_Se)]
*M* *_r_* = 351.85
Orthorhombic, 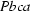

*a* = 13.0352 (12) Å
*b* = 10.0882 (13) Å
*c* = 18.709 (2) Å
*V* = 2460.3 (5) Å^3^
*Z* = 8
Mo *K*α radiation
μ = 5.01 mm^−1^
*T* = 298 K
0.42 × 0.33 × 0.29 mm

#### Data collection

Bruker SMART 1000 CCD area-detector diffractometer
Absorption correction: multi-scan (*SADABS*; Sheldrick, 1996 ▶) *T*_min_ = 0.228, *T*_max_ = 0.325
9382 measured reflections
2164 independent reflections
1681 reflections with *I* > 2σ(*I*)
*R*_int_ = 0.048

#### Refinement

*R*[*F* ^2^ > 2σ(*F* ^2^)] = 0.028
*wR*(*F* ^2^) = 0.069
*S* = 1.06
2164 reflections
118 parameters
H-atom parameters constrained
Δρ_max_ = 0.39 e Å^−3^
Δρ_min_ = −0.45 e Å^−3^

### 

Data collection: *SMART* (Siemens, 1996 ▶); cell refinement: *SAINT* (Siemens, 1996 ▶); data reduction: *SAINT*; program(s) used to solve structure: *SHELXS97* (Sheldrick, 2008 ▶); program(s) used to refine structure: *SHELXL97* (Sheldrick, 2008 ▶); molecular graphics: *SHELXTL* (Sheldrick, 2008 ▶); software used to prepare material for publication: *SHELXTL*.

## Supplementary Material

Crystal structure: contains datablocks I, global. DOI: 10.1107/S1600536810054243/rz2544sup1.cif

Structure factors: contains datablocks I. DOI: 10.1107/S1600536810054243/rz2544Isup2.hkl

Additional supplementary materials:  crystallographic information; 3D view; checkCIF report

## Figures and Tables

**Fig. 1.**
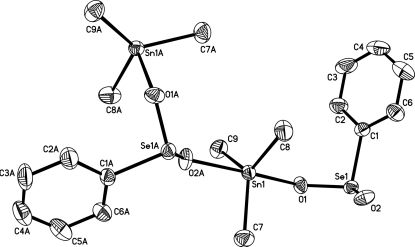
The coordination geometry of the tin metal centre in the title compound, showing 50% probability displacement ellipsoids. H atoms are omitted for clarity. [Symmetry code: (A) -x, -1/2+y, 1/2-z]

**Fig. 2.**
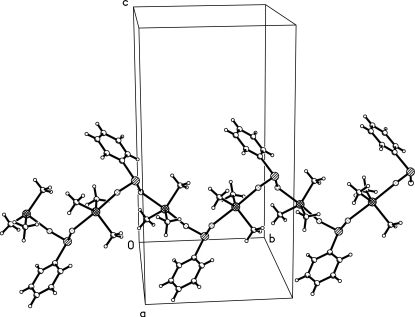
View of the one-dimensional zigzag chain structure running parallel to the b axis in the title compound.

## supplementary crystallographic information

### Comment

In recent years, organotin compounds have been attracting more and more attention due to their wide range of industrial applications and biological activities (Dubey & Roy, 2003). As a part of our ongoing investigations in this field, we have synthesized the title compound and present its crystal structure here.

The asymmetric unit of the title compound is shown in Fig. 1. An extended one-dimensional zigzag chain structure running parallel to the *b* axis is formed by the bridging role of the phenylseleninato anions (Fig. 2). The Sn—O bond distances in the compound (Sn1—O1 = 2.243 (3) Å; Sn1—O2^i^ = 2.258 (3) Å; symmetry code: (i): -*x*, *y*-1/2, -*z*+1/2) are comparable to those found in a related organotin compound (Chandrasekhar *et al.*, 1992). The Sn atom is five-coordinate in a slightly distorted trigonal-bipyramidal coordination geometry, provided by the methyl groups in the equatorial positions and two O atoms of symmetry related phenylseleninato groups in the axial positions.

### Experimental

The reaction was carried out under a nitrogen atmosphere. Benzeneseleninic acid (1 mmol) and sodium ethoxide (1 mmol) were added to a stirred solution of methanol (30 ml) in a Schlenk flask and stirred for 0.5 h. Trimethyltin chloride (1 mmol) was then added to the reactor and the reaction mixture was stirred for 12 h at at room temperature. The resulting clear solution was evaporated under vacuum. The product was crystallized from a solution of ether to yield colourless block crystals of the title compound (yield 65%). Anal. Calcd (%) for C_9_H_14_O_2_Sn_1_Se_1_ (Mr = 351.85): C, 30.72; H, 4.01. Found (%): C, 30.93; H, 3.85.

### Refinement

The H atoms were positioned geometrically, with methyl C—H distances of 0.96Å and aromatic C—H distances of 0.93 Å, and refined as riding on their parent atoms, with *U*_iso_(H) = 1.2 *U*_eq_(C) or 1.5 *U*_eq_(C) for the methyl groups.

### Crystal data

**Table:**

[Sn(CH_3_)_3_(C_6_H_5_O_2_Se)]	*F*(000) = 1344
*M**_r_* = 351.85	*D*_x_ = 1.900 Mg m^−^^3^
Orthorhombic, *P**b**c**a*	Mo *K*α radiation, λ = 0.71073 Å
Hall symbol: -P 2ac 2ab	Cell parameters from 3552 reflections
*a* = 13.0352 (12) Å	θ = 2.7–26.4°
*b* = 10.0882 (13) Å	µ = 5.01 mm^−^^1^
*c* = 18.709 (2) Å	*T* = 298 K
*V* = 2460.3 (5) Å^3^	Block, colourless
*Z* = 8	0.42 × 0.33 × 0.29 mm

### Data collection

**Table:**

Bruker SMART 1000 CCD area-detector diffractometer	2164 independent reflections
Radiation source: fine-focus sealed tube	1681 reflections with *I* > 2σ(*I*)
graphite	*R*_int_ = 0.048
phi and ω scans	θ_max_ = 25.0°, θ_min_ = 2.2°
Absorption correction: multi-scan (*SADABS*; Sheldrick, 1996)	*h* = −8→15
*T*_min_ = 0.228, *T*_max_ = 0.325	*k* = −12→11
9382 measured reflections	*l* = −22→22

### Refinement

**Table:**

Refinement on *F*^2^	Primary atom site location: structure-invariant direct methods
Least-squares matrix: full	Secondary atom site location: difference Fourier map
*R*[*F*^2^ > 2σ(*F*^2^)] = 0.028	Hydrogen site location: inferred from neighbouring sites
*wR*(*F*^2^) = 0.069	H-atom parameters constrained
*S* = 1.06	*w* = 1/[σ^2^(*F*_o_^2^) + (0.0321*P*)^2^ + 0.1006*P*] where *P* = (*F*_o_^2^ + 2*F*_c_^2^)/3
2164 reflections	(Δ/σ)_max_ = 0.001
118 parameters	Δρ_max_ = 0.39 e Å^−^^3^
0 restraints	Δρ_min_ = −0.45 e Å^−^^3^

### Fractional atomic coordinates and isotropic or equivalent isotropic displacement parameters (Å^2^)

**Table:**

	*x*	*y*	*z*	*U*_iso_*/*U*_eq_	
Sn1	−0.06314 (2)	0.18100 (3)	0.263140 (17)	0.03827 (12)	
Se1	−0.03615 (4)	0.46755 (5)	0.37539 (2)	0.03951 (15)	
O1	−0.1012 (2)	0.3364 (3)	0.34469 (17)	0.0485 (8)	
O2	0.0495 (3)	0.5076 (3)	0.31255 (19)	0.0568 (9)	
C1	0.0573 (4)	0.3775 (5)	0.4398 (2)	0.0401 (11)	
C2	0.0239 (5)	0.2699 (6)	0.4774 (3)	0.0612 (15)	
H2	−0.0433	0.2404	0.4724	0.073*	
C3	0.0902 (6)	0.2054 (6)	0.5228 (3)	0.078 (2)	
H3	0.0680	0.1315	0.5482	0.094*	
C4	0.1899 (6)	0.2502 (7)	0.5309 (3)	0.075 (2)	
H4	0.2345	0.2070	0.5619	0.090*	
C5	0.2223 (5)	0.3571 (7)	0.4933 (3)	0.0740 (18)	
H5	0.2893	0.3873	0.4986	0.089*	
C6	0.1567 (4)	0.4208 (6)	0.4477 (3)	0.0552 (14)	
H6	0.1795	0.4937	0.4218	0.066*	
C7	−0.1262 (5)	0.2998 (5)	0.1810 (3)	0.0647 (16)	
H7A	−0.1806	0.3532	0.2002	0.097*	
H7B	−0.1528	0.2438	0.1439	0.097*	
H7C	−0.0738	0.3562	0.1616	0.097*	
C8	0.0976 (4)	0.1852 (6)	0.2800 (3)	0.0678 (17)	
H8A	0.1295	0.1162	0.2525	0.102*	
H8B	0.1119	0.1716	0.3298	0.102*	
H8C	0.1243	0.2696	0.2654	0.102*	
C9	−0.1570 (4)	0.0505 (5)	0.3233 (3)	0.0614 (15)	
H9A	−0.1983	0.1008	0.3560	0.092*	
H9B	−0.1144	−0.0100	0.3496	0.092*	
H9C	−0.2007	0.0016	0.2916	0.092*	

### Atomic displacement parameters (Å^2^)

**Table:**

	*U*^11^	*U*^22^	*U*^33^	*U*^12^	*U*^13^	*U*^23^
Sn1	0.03808 (19)	0.0389 (2)	0.0379 (2)	−0.00017 (15)	−0.00099 (14)	−0.00287 (15)
Se1	0.0485 (3)	0.0345 (3)	0.0356 (3)	0.0079 (2)	−0.0021 (2)	−0.0026 (2)
O1	0.0439 (18)	0.050 (2)	0.052 (2)	0.0053 (16)	−0.0044 (16)	−0.0186 (16)
O2	0.068 (2)	0.048 (2)	0.054 (2)	0.0072 (17)	0.0061 (19)	0.0209 (17)
C1	0.051 (3)	0.040 (3)	0.029 (2)	0.006 (2)	0.001 (2)	0.000 (2)
C2	0.062 (4)	0.064 (4)	0.058 (4)	0.000 (3)	−0.006 (3)	0.013 (3)
C3	0.115 (6)	0.065 (4)	0.056 (4)	0.016 (4)	−0.009 (4)	0.025 (3)
C4	0.087 (5)	0.086 (5)	0.052 (4)	0.041 (4)	−0.016 (3)	−0.003 (4)
C5	0.054 (4)	0.109 (6)	0.059 (4)	0.012 (3)	−0.014 (3)	−0.004 (4)
C6	0.056 (3)	0.068 (4)	0.042 (3)	−0.002 (3)	−0.003 (3)	0.002 (3)
C7	0.085 (4)	0.061 (4)	0.048 (3)	0.004 (3)	−0.003 (3)	0.009 (3)
C8	0.038 (3)	0.068 (4)	0.097 (5)	0.012 (3)	−0.006 (3)	−0.028 (3)
C9	0.076 (4)	0.056 (4)	0.052 (3)	−0.006 (3)	0.010 (3)	0.004 (3)

### Geometric parameters (Å, °)

**Table:**

Sn1—C7	2.115 (5)	C4—C5	1.355 (9)
Sn1—C8	2.119 (5)	C4—H4	0.9300
Sn1—C9	2.121 (5)	C5—C6	1.368 (7)
Sn1—O1	2.243 (3)	C5—H5	0.9300
Sn1—O2^i^	2.258 (3)	C6—H6	0.9300
Se1—O2	1.671 (3)	C7—H7A	0.9600
Se1—O1	1.673 (3)	C7—H7B	0.9600
Se1—C1	1.940 (5)	C7—H7C	0.9600
O2—Sn1^ii^	2.258 (3)	C8—H8A	0.9600
C1—C2	1.365 (7)	C8—H8B	0.9600
C1—C6	1.375 (7)	C8—H8C	0.9600
C2—C3	1.376 (8)	C9—H9A	0.9600
C2—H2	0.9300	C9—H9B	0.9600
C3—C4	1.384 (9)	C9—H9C	0.9600
C3—H3	0.9300		
			
C7—Sn1—C8	118.8 (3)	C3—C4—H4	120.1
C7—Sn1—C9	120.9 (2)	C4—C5—C6	120.2 (6)
C8—Sn1—C9	120.2 (2)	C4—C5—H5	119.9
C7—Sn1—O1	90.72 (18)	C6—C5—H5	119.9
C8—Sn1—O1	95.92 (17)	C5—C6—C1	120.4 (6)
C9—Sn1—O1	86.86 (17)	C5—C6—H6	119.8
C7—Sn1—O2^i^	90.78 (18)	C1—C6—H6	119.8
C8—Sn1—O2^i^	91.79 (18)	Sn1—C7—H7A	109.5
C9—Sn1—O2^i^	84.11 (17)	Sn1—C7—H7B	109.5
O1—Sn1—O2^i^	170.25 (12)	H7A—C7—H7B	109.5
O2—Se1—O1	106.75 (18)	Sn1—C7—H7C	109.5
O2—Se1—C1	97.51 (18)	H7A—C7—H7C	109.5
O1—Se1—C1	99.28 (18)	H7B—C7—H7C	109.5
Se1—O1—Sn1	132.40 (18)	Sn1—C8—H8A	109.5
Se1—O2—Sn1^ii^	132.94 (18)	Sn1—C8—H8B	109.5
C2—C1—C6	119.9 (5)	H8A—C8—H8B	109.5
C2—C1—Se1	119.5 (4)	Sn1—C8—H8C	109.5
C6—C1—Se1	120.7 (4)	H8A—C8—H8C	109.5
C1—C2—C3	119.6 (6)	H8B—C8—H8C	109.5
C1—C2—H2	120.2	Sn1—C9—H9A	109.5
C3—C2—H2	120.2	Sn1—C9—H9B	109.5
C2—C3—C4	120.2 (6)	H9A—C9—H9B	109.5
C2—C3—H3	119.9	Sn1—C9—H9C	109.5
C4—C3—H3	119.9	H9A—C9—H9C	109.5
C5—C4—C3	119.7 (6)	H9B—C9—H9C	109.5
C5—C4—H4	120.1		
			
O2—Se1—O1—Sn1	22.8 (3)	O1—Se1—C1—C6	143.5 (4)
C1—Se1—O1—Sn1	−78.0 (3)	C6—C1—C2—C3	−0.2 (8)
C7—Sn1—O1—Se1	−89.2 (3)	Se1—C1—C2—C3	179.6 (4)
C8—Sn1—O1—Se1	29.8 (3)	C1—C2—C3—C4	0.7 (9)
C9—Sn1—O1—Se1	149.9 (3)	C2—C3—C4—C5	−0.6 (10)
O1—Se1—O2—Sn1^ii^	110.1 (3)	C3—C4—C5—C6	0.1 (9)
C1—Se1—O2—Sn1^ii^	−147.8 (3)	C4—C5—C6—C1	0.4 (8)
O2—Se1—C1—C2	−144.7 (4)	C2—C1—C6—C5	−0.4 (8)
O1—Se1—C1—C2	−36.2 (4)	Se1—C1—C6—C5	179.8 (4)
O2—Se1—C1—C6	35.1 (4)		

Symmetry codes: (i) −*x*, *y*−1/2, −*z*+1/2; (ii) −*x*, *y*+1/2, −*z*+1/2.
